# Antagonism of the Sodium-Potassium ATPase Impairs Chikungunya Virus Infection

**DOI:** 10.1128/mBio.00693-16

**Published:** 2016-05-24

**Authors:** Alison W. Ashbrook, Anthony J. Lentscher, Paula F. Zamora, Laurie A. Silva, Nicholas A. May, Joshua A. Bauer, Thomas E. Morrison, Terence S. Dermody

**Affiliations:** a Department of Pediatrics, Vanderbilt University School of Medicine, Nashville, Tennessee, USA; b Elizabeth B. Lamb Center for Pediatric Research, Vanderbilt University School of Medicine, Nashville, Tennessee, USA; c Department of Pathology, Microbiology, and Immunology, Vanderbilt University School of Medicine, Nashville, Tennessee, USA; d Department of Immunology and Microbiology, University of Colorado School of Medicine, Aurora, Colorado, USA; e Department of Biochemistry, Vanderbilt University School of Medicine, Nashville, Tennessee, USA; f Vanderbilt Institute of Chemical Biology, High-Throughput Screening Facility, Vanderbilt University School of Medicine, Nashville, Tennessee, USA

## Abstract

Chikungunya virus (CHIKV) is a reemerging alphavirus that has caused epidemics of fever, arthralgia, and rash worldwide. There are currently no licensed vaccines or antiviral therapies available for the prevention or treatment of CHIKV disease. We conducted a high-throughput, chemical compound screen that identified digoxin, a cardiac glycoside that blocks the sodium-potassium ATPase, as a potent inhibitor of CHIKV infection. Treatment of human cells with digoxin or a related cardiac glycoside, ouabain, resulted in a dose-dependent decrease in infection by CHIKV. Inhibition by digoxin was cell type-specific, as digoxin treatment of either murine or mosquito cells did not diminish CHIKV infection. Digoxin displayed antiviral activity against other alphaviruses, including Ross River virus and Sindbis virus, as well as mammalian reovirus and vesicular stomatitis virus. The digoxin-mediated block to CHIKV and reovirus infection occurred at one or more postentry steps, as digoxin inhibition was not bypassed by fusion of CHIKV at the plasma membrane or infection with cell surface-penetrating reovirus entry intermediates. Selection of digoxin-resistant CHIKV variants identified multiple mutations in the nonstructural proteins required for replication complex formation and synthesis of viral RNA. These data suggest a role for the sodium-potassium ATPase in promoting postentry steps of CHIKV replication and provide rationale for modulation of this pathway as a broad-spectrum antiviral strategy.

## IMPORTANCE

Mitigation of disease induced by globally spreading, mosquito-borne arthritogenic alphaviruses requires the development of new antiviral strategies. High-throughput screening of clinically tested compounds provides a rapid means to identify undiscovered, antiviral functions for well-characterized therapeutics and illuminate host pathways required for viral infection. Our study describes the potent inhibition of CHIKV and related alphaviruses by the cardiac glycoside digoxin and demonstrates a function for the sodium-potassium ATPase in CHIKV infection.

## INTRODUCTION

Chikungunya virus (CHIKV) is an arthritogenic alphavirus responsible for explosive epidemics throughout the world. Since its reemergence in Kenya in 2004, millions of cases of CHIKV have been reported in sub-Saharan Africa and Asia in addition to regions in which CHIKV was not previously endemic, including Europe and the Americas (1–6). Autochthonous, mosquito transmission of CHIKV continues to occur in many countries of the Caribbean basin and South America, and the presence of CHIKV-competent mosquito vectors in these regions supports the potential for further spread of the virus to new populations.

The vast majority of CHIKV-infected individuals develop chikungunya fever, a disease characterized by debilitating polyarthralgia and arthritis, headache, and rash (7, 8). More severe disease and atypical symptoms also have been observed during recent epidemics (9–11), including neurological and cardiac manifestations, which have been reported in neonates, the elderly, and those with underlying comorbidities. Although most of the clinical signs and symptoms resolve 7 to 10 days after infection, the arthritis and polyarthralgia can recur for months to years after the initial diagnosis (2, 8, 12). To date, no licensed anti-CHIKV therapeutics or vaccines are available. The chronic, incapacitating disease, in addition to the high attack rates of the virus in naive populations, imposes a substantial burden on the quality of life of those infected and the economies of affected countries (13–15).

CHIKV displays broad tropism in humans, but many of the host factors required for infection are not fully understood. Following attachment to host cells via unidentified cell surface receptors, CHIKV particles are internalized by clathrin-mediated endocytosis (16–18). Acidification of endosomes triggers fusion of the viral envelope with the host endosomal membrane, which allows release of the nucleocapsid into the cytoplasm (19, 20). The CHIKV genome consists of a single-stranded, positive-sense RNA molecule approximately 12 kb in length that encodes four nonstructural proteins (nsP1 to 4) and three major structural proteins (capsid, pE2, and E1) (21, 22). Together, the nonstructural proteins mediate interactions with cellular membranes and other host factors to form replication complexes that house synthesis of subgenomic RNA and additional copies of viral genomic RNA for encapsidation into progeny virions (23–28). Host factors and mechanisms involved in viral RNA synthesis of alphaviruses, particularly for CHIKV, are not well defined.

To identify host mediators of CHIKV replication, we screened a library of small molecules for the capacity to augment or diminish infection of human osteosarcoma (U-2 OS) cells by CHIKV replicon particles expressing an enhanced green fluorescent protein (eGFP) reporter. From this screen, we identified digoxin, a cardiac glycoside that antagonizes the sodium-potassium ATPase, as a potent inhibitor of CHIKV infection. Digoxin diminished infection by replication-competent CHIKV in both U-2 OS cells and primary human synovial fibroblasts. Ouabain, a related cardiac glycoside, also blocked CHIKV infection. Increasing extracellular concentrations of potassium alleviated CHIKV inhibition by digoxin, suggesting that antagonism of the sodium-potassium ATPase mediates the antiviral effect. Digoxin displayed antiviral activity against alphaviruses other than CHIKV, including Ross River virus (RRV) and Sindbis virus (SINV), and the unrelated mammalian orthoreovirus (called “reovirus” here) and vesicular stomatitis virus (VSV). Passage of CHIKV in digoxin-treated cells selected mutations in genes encoding nonstructural proteins, suggesting that digoxin impairs functions mediated by these replicase proteins. These data suggest a role for the sodium-potassium ATPase in CHIKV infection and highlight a new strategy for development of therapeutics to limit CHIKV replication and disease.

## RESULTS

### Identification of digoxin as an inhibitor of CHIKV infection.

**FIG 1:**
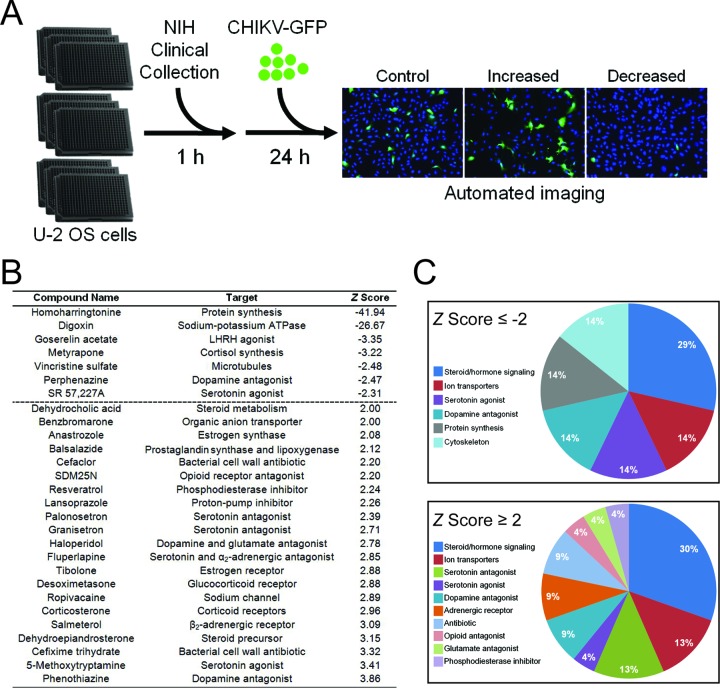
High-throughput screening to identify inhibitors of CHIKV infection. (A) U-2 OS cells were incubated with DMSO, 100 nM bafilomycin A1, or compounds from the NIH clinical collection at a concentration of 1 µM at 37°C for 1 h. Cells were adsorbed with SL15649 eGFP replicon particles at an MOI of ~5 IU/cell and incubated with compound at 37°C for 20 to 24 h. Cells were incubated with Hoechst dye to stain nuclei and imaged by automated, high-content fluorescence microscopy. (B) Robust *Z* scores were calculated for individual compounds. Shown are the average robust *Z* scores for compounds with robust *Z* scores of ≤−2 or ≥2 median absolute deviations from the median of each plate identified in three independent screening experiments. (C) Distribution of candidate compounds by known biological targets.

To identify host factors required for CHIKV infection, we screened 727 chemical compounds from the NIH Clinical Collection (NCC) for the capacity to impede or augment infection by CHIKV replicon particles (Fig. 1A). The NCC library consists almost entirely of compounds that have been used in phase I, II, and III clinical trials. U-2 OS cells were incubated with dimethyl sulfoxide (DMSO) as a vehicle control, 100 nM bafilomycin A1 as a positive control, or 1 µM NCC compound for 1 h. Treated cells were adsorbed with CHIKV strain SL15649 replicon particles expressing eGFP and incubated for 20 to 24 h. The percentage of infected cells was determined by GFP expression, and robust *Z* scores were calculated for each compound from three independent experiments. Seven compounds had average *Z* scores of ≤−2.0 (inhibited infection), and 21 compounds had average *Z* scores of ≥2.0 (enhanced infection) (Fig. 1B). The largest class of compounds that influenced CHIKV infection, both positively and negatively, is that which affects steroid or hormone signaling and biosynthetic pathways (Fig. 1C). CHIKV infection was also positively and negatively affected by compounds that target ion transporters and neurotransmitter receptors, such as dopamine and serotonin. Homoharringtonine, a translation inhibitor and known antagonist of CHIKV infection (29), had the largest negative *Z* score, −41.94. Digoxin, a sodium-potassium ATPase inhibitor, had a negative *Z* score of −26.67, suggesting a function for the sodium-potassium ATPase in CHIKV infection.

### Digoxin is a species-specific inhibitor of CHIKV infection.

**FIG 2:**
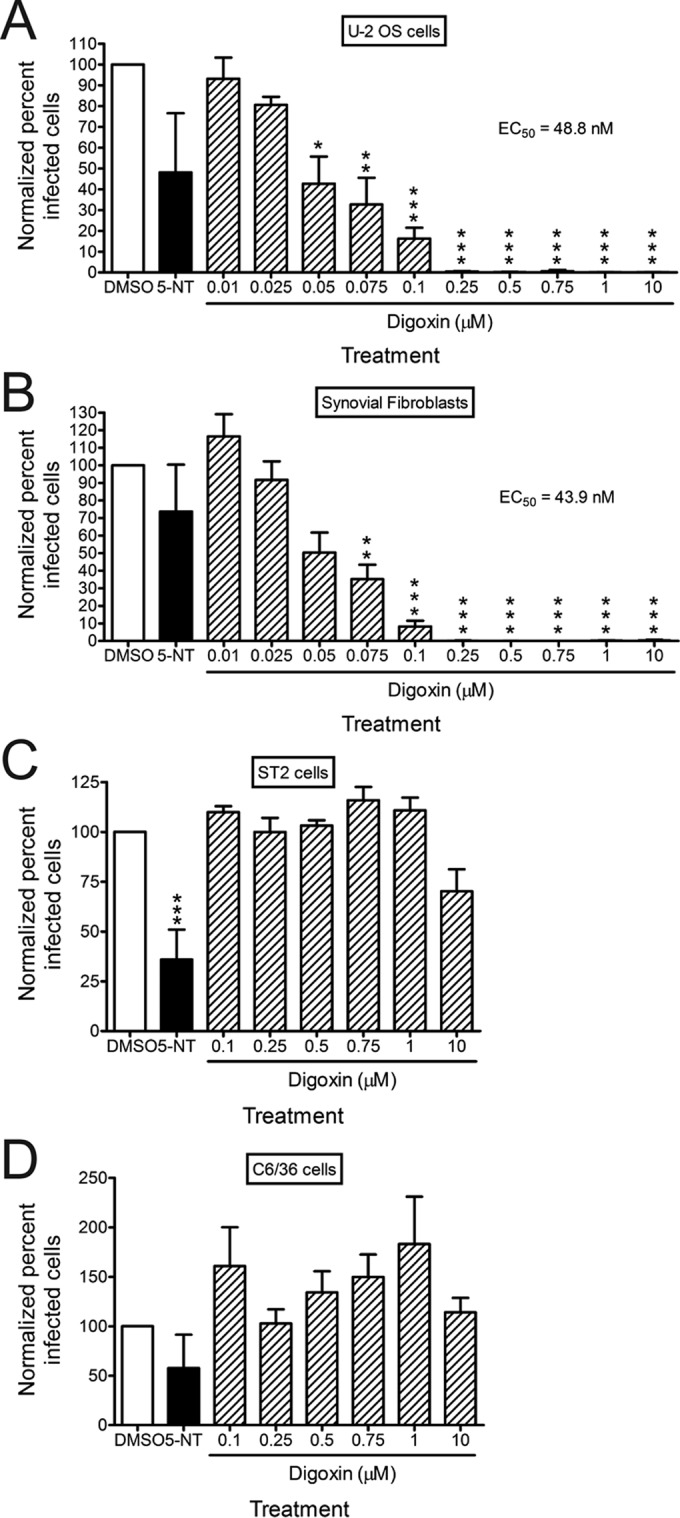
Digoxin potently inhibits CHIKV infection of human cells. (A) U-2 OS cells, (B) HSFs, (C) ST2 cells, or (D) C6/36 cells were incubated with DMSO, 10 µM 5-NT, or increasing concentrations of digoxin for 1 h prior to adsorption with CHIKV strain SL15649 at an MOI of 5 PFU/cell. After 1 h of incubation, virus was removed, and cells were incubated with medium containing DMSO or inhibitor for 5 h. Cells were stained with CHIKV-specific antiserum and DAPI to detect nuclei and imaged by fluorescence microscopy. Results are presented as percentages of infected cells normalized to DMSO-treated cells for triplicate experiments. Error bars indicate standard errors of the means. *, *P* < 0.05, **, *P* < 0.01, and ***, *P* < 0.001, in comparison to DMSO, as determined by ANOVA followed by Tukey’s post hoc test.

To determine whether inhibition of the sodium-potassium ATPase blocks infection by replication-competent CHIKV, we treated a variety of cell lines with DMSO, 5-nonyloxytryptamine (5-NT [a serotonin receptor agonist]) as a positive control (30), or increasing concentrations of digoxin for 1 h prior to adsorption with CHIKV. At 6 h postinfection, cells were scored for infection by indirect immunofluorescence (Fig. 2). Relative to DMSO-treated cells, treatment of U-2 OS cells with digoxin resulted in a dose-dependent decrease in CHIKV infection with a half-maximal effective concentration (EC_50_) of 48.8 nM (Fig. 2A). Digoxin treatment similarly decreased CHIKV infection of primary human synovial fibroblasts (HSFs) and Vero African green monkey kidney cells with EC_50_s of 43.9 nM and 67.3 nM, respectively (Fig. 2B and data not shown). Incubation of virus with digoxin prior to adsorption to cells had no effect on viral titers, suggesting that the antiviral effect is not attributable to an alteration in the virus (data not shown). Despite inhibition of CHIKV infection in multiple primate cell types, digoxin treatment of murine stromal ST2 and *Aedes albopictus* C6/36 cells at doses sufficient to block infection of primate cells did not decrease CHIKV infection (Fig. 2C and D). Together, these data indicate that digoxin is a potent inhibitor of CHIKV infection and that inhibition occurs in a host species-specific manner.

### Species-specific inhibition by digoxin occurs via the sodium-potassium ATPase.

**FIG 3:**
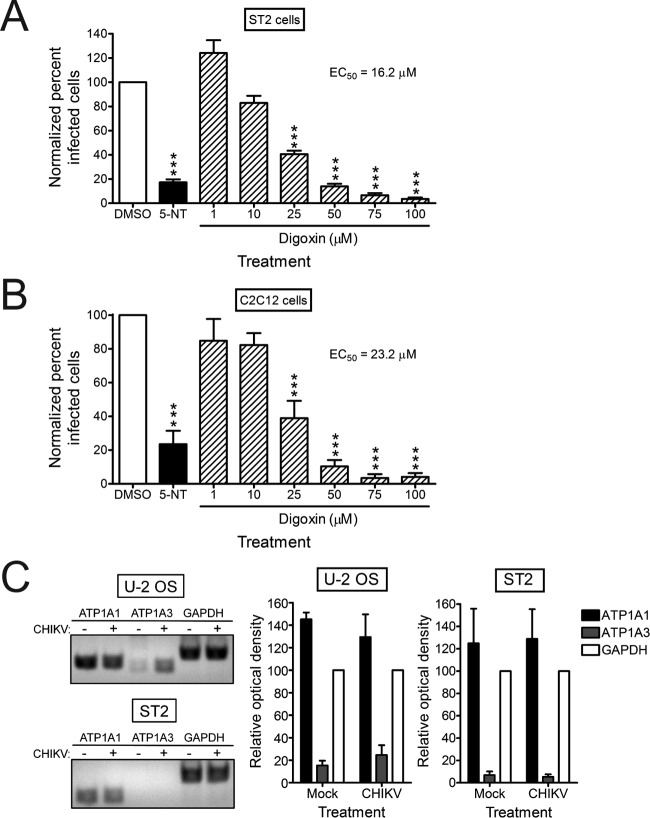
CHIKV resistance to digoxin in murine cells correlates with decreased expression of the α3 subunit of the sodium-potassium ATPase. (A) ST2 cells or (B) C2C12 cells were incubated with DMSO, 10 µM 5-NT, or increasing concentrations of digoxin for 1 h prior to adsorption with CHIKV strain SL15649 at an MOI of 5 PFU/cell. After 1 h, virus was removed, and cells were incubated with medium containing DMSO or inhibitor for 5 h. Cells were stained with CHIKV-specific antiserum and DAPI to detect nuclei and imaged by fluorescence microscopy. Results are presented as the percentages of infected cells normalized to DMSO-treated cells for triplicate experiments. Error bars indicate standard errors of the means. (C) U-2 OS and ST2 cells were mock infected or infected with CHIKV 181/25 at an MOI of 5 PFU/cell. RNA was isolated and used for RT-PCR amplification of ATP1A1 (α1), ATP1A3 (α3), and GAPDH transcripts with human- or murine-specific primer sets (see Table S1 in the supplemental material). Reaction products were resolved by electrophoresis in 1% agarose gels (left). Band intensity was quantified by optical densitometry for four independent experiments (right). ***, *P* < 0.001, in comparison to DMSO, as determined by ANOVA followed by Tukey’s post hoc test.

Cardiac glycosides inhibit the sodium-potassium ATPase by binding to the catalytic α subunit but do so with less efficiency to specific murine isoforms relative to their human counterparts (31–33). To determine whether higher doses of digoxin are capable of inhibiting CHIKV infection of murine cells, ST2 cells and myoblast C2C12 cells were treated with DMSO, 5-NT, or increasing concentrations of digoxin for 1 h prior to adsorption with CHIKV. At 6 h postinfection, cells were scored for infection by indirect immunofluorescence (Fig. 3A and B). At higher concentrations, digoxin treatment significantly diminished CHIKV infection in these cell types, with EC_50_s of 16.2 µM in ST2 cells and 23.2 µM in C2C12 cells, values 330 and 475 times the EC_50_ of digoxin in U-2 OS cells, without a decrease in cell viability (data not shown). These data indicate that digoxin can inhibit CHIKV infection of murine cells, but at significantly higher concentrations than in human cells.

We next assessed transcript levels of two α subunit isoforms (α1 and α3) in the human and murine cells tested. Whereas the human and murine α3 subunits are sensitive to cardiac glycosides, the murine α1 isoform is significantly less sensitive to cardiac glycoside treatment relative to the human isoform (31, 32). RNA was isolated from mock-infected and CHIKV-infected U-2 OS and ST2 cells and used as a template for reverse transcription-PCR (RT-PCR) amplification of ATP1A1 (α1), ATP1A3 (α3), and GAPDH (glyceraldehyde-3-phosphate dehydrogenase) (as a control) mRNAs (Fig. 3C). Expression of ATP1A1 was detected in both U-2 OS and ST2 cells and did not differ significantly following infection. In contrast, the ATP1A3 transcript was detected in U-2 OS cells but not in ST2 cells. Thus, decreased expression of ATP1A3 in murine cells correlates with reduced sensitivity to digoxin-mediated inhibition of CHIKV.

**FIG 4:**
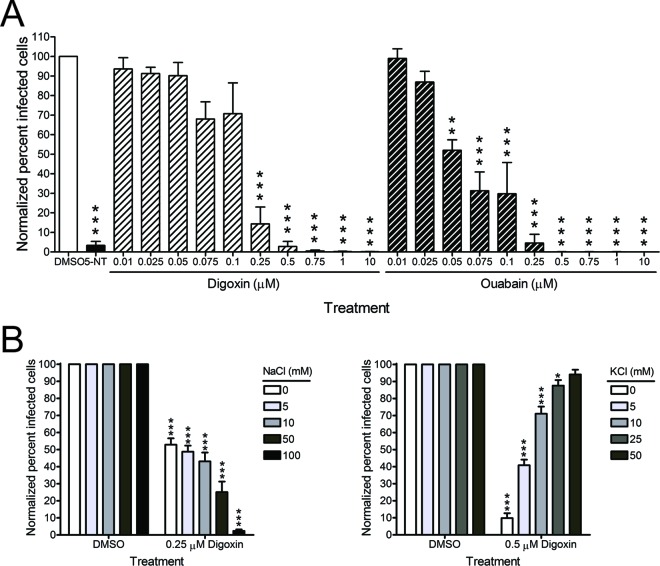
Inhibition by digoxin occurs via the sodium-potassium ATPase. (A) U-2 OS cells were incubated with DMSO, 10 µM 5-NT, or increasing concentrations of digoxin or the related cardiac glycoside, ouabain, for 1 h prior to adsorption with CHIKV SL15649 at an MOI of 5 PFU/cell. After 1 h, virus was removed, and cells were incubated with medium containing DMSO or inhibitor for 5 h. Cells were scored for infection by indirect immunofluorescence. Results are presented as percentage of infected cells normalized to DMSO-treated cells for triplicate experiments. Error bars indicate standard errors of the means. (B) U-2 OS cells were incubated with DMSO or digoxin in standard medium or medium supplemented with increasing concentrations of NaCl (left) or KCl (right) for 1 h prior to adsorption with CHIKV 181/25 at an MOI of 5 PFU/cell. After 1 h, virus was removed, and cells were incubated with medium containing DMSO or digoxin and the concentration of NaCl or KCl shown for 5 h. Cells were scored for infection by indirect immunofluorescence. Results are presented as percentages of infected cells normalized to DMSO-treated cells for triplicate experiments. Error bars indicate standard errors of the means. *, *P* < 0.05, **, *P* < 0.01, and ***, *P* < 0.001, in comparison to DMSO, as determined by ANOVA followed by Tukey’s post hoc test.

To determine whether blockade of the sodium-potassium ATPase is responsible for CHIKV inhibition, U-2 OS cells were treated with increasing concentrations of digoxin or ouabain, a related cardiac glycoside, for 1 h prior to adsorption with CHIKV. At 6 h postinfection, cells were scored for infection by indirect immunofluorescence (Fig. 4A). Again, treatment of cells with ouabain led to a dose-dependent decrease in CHIKV infection, indicating that treatment of cells with two independent cardiac glycosides results in decreased CHIKV infection. As the sodium-potassium ATPase is the only known target of cardiac glycosides, these data suggest that antagonism of this molecule is the mechanism by which digoxin restricts CHIKV. To further test whether inhibition of CHIKV by digoxin treatment occurs via changes in ion concentrations, cells were pretreated with DMSO or digoxin in the presence of increasing extracellular sodium or potassium for 1 h prior to adsorption with CHIKV. At 6 h postinfection, cells were scored for infection by indirect immunofluorescence (Fig. 4B). Addition of extracellular NaCl in the presence of digoxin enhanced inhibition of CHIKV by digoxin in a dose-dependent manner (Fig. 4B, left). Furthermore, addition of extracellular KCl in the presence of digoxin alleviated inhibition of CHIKV by digoxin (Fig. 4B, right). These data indicate that alterations of ion concentrations contribute to the antiviral activity of digoxin.

### CHIKV inhibition by digoxin is not attributable to decreased cell viability.

**FIG 5:**
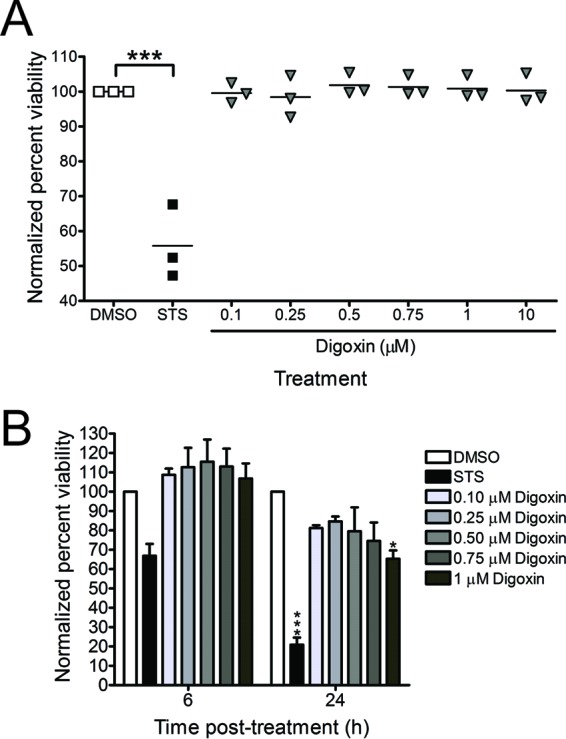
CHIKV inhibition by digoxin is not attributable to cytotoxicity. (A) U-2 OS cells were treated with DMSO, 10 µM STS, or increasing concentrations of digoxin for 6 h. Cell viability was quantified by PI staining. Results are expressed as percentages of viable cells normalized to DMSO-treated cells for individual experiments. Horizontal black lines indicate mean percentages of viability. (B) U-2 OS cells were treated with DMSO, 10 µM STS, or increasing concentrations of digoxin for 6 or 24 h. Cell viability was quantified by PrestoBlue fluorescence assay. Results are presented as percentages of viable cells normalized to DMSO-treated cells for triplicate experiments. Error bars indicate standard errors of the means. *, *P* < 0.05, and ***, *P* < 0.001, in comparison to DMSO, as determined by ANOVA followed by Tukey’s post hoc test.

The sodium-potassium ATPase is essential for homeostasis in most multicellular organisms. As such, we sought to determine whether inhibition of CHIKV infection by digoxin occurs as a consequence of altered viability of treated cells. To assess possible digoxin cytotoxicity, cells were incubated with either propidium iodide (PI) or PrestoBlue to assess plasma membrane integrity and mitochondrial metabolic activity, respectively, posttreatment with DMSO, staurosporine (STS) (as an inducer of cell death), or increasing concentrations of digoxin (Fig. 5). Incubation of U-2 OS cells with digoxin did not alter cell viability by 6 h posttreatment (Fig. 5A and B). Moreover, cell viability was only modestly impaired at 24 h posttreatment with 1 µM digoxin, a dose 20 times the digoxin EC_50_ for CHIKV antiviral activity in these cells (Fig. 5B). Digoxin also did not significantly decrease viability of HSFs and Vero cells following treatment with doses of digoxin that inhibit CHIKV infection in these cells (data not shown). These data suggest that digoxin inhibition of CHIKV is not attributable to cytotoxic effects.

### Digoxin treatment inhibits infection by diverse virus families.

**FIG 6:**
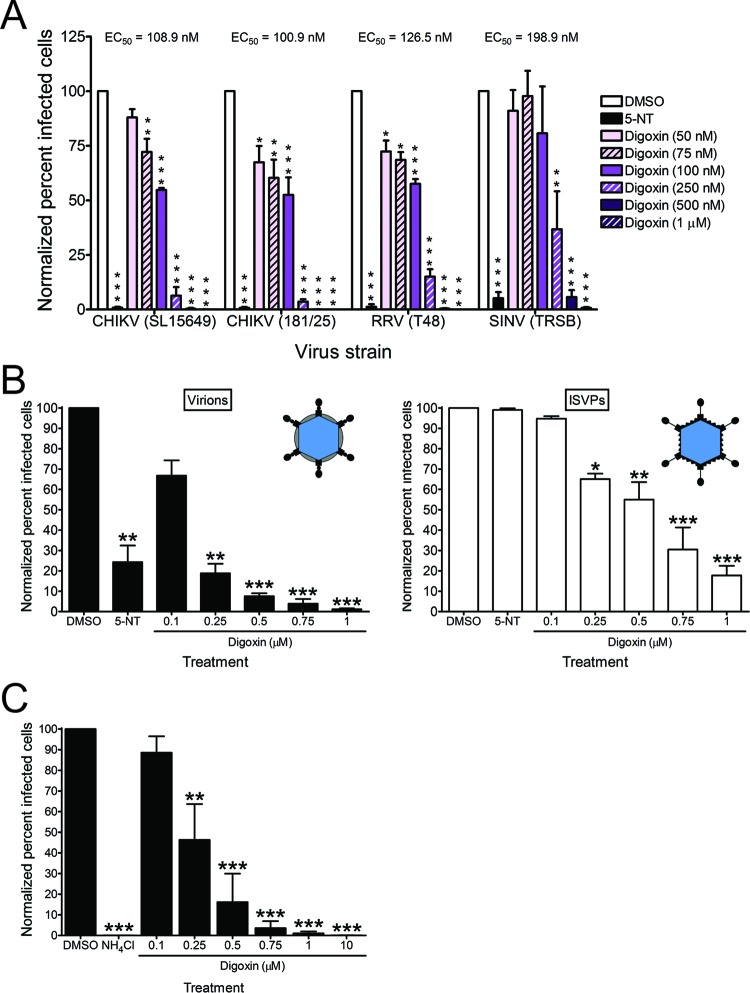
Digoxin inhibits multiple alphaviruses, mammalian reovirus, and VSV. (A) U-2 OS cells were incubated with DMSO, 10 µM 5-NT, or increasing concentrations of digoxin for 1 h prior to adsorption with CHIKV strains SL15649 and 181/25 at an MOI of 1 PFU/cell, RRV strain T48 at an MOI of 10 PFU/cell, or SINV strain TRSB at an MOI of 5 PFU/cell. After 1 h of incubation, virus was removed, and cells were incubated with medium containing DMSO or inhibitor for 5 h. Cells were stained with virus-specific antiserum and DAPI to detect nuclei and imaged by fluorescence microscopy. Results are presented as percentages of infected cells normalized to DMSO-treated cells for triplicate experiments. Error bars indicate standard errors of the mean. (B) HBMECs were incubated with DMSO, 10 µM 5-NT, or increasing concentrations of digoxin for 1 h prior to adsorption with reovirus virions (left) or ISVPs (right) at an MOI of 1,500 particles/cell (~15 PFU/cell). After 1 h of incubation, virus was removed, and cells were incubated with medium containing DMSO or inhibitor for 20 h. Cells were scored for infection by indirect immunofluorescence. Results are presented as percentages of infected cells normalized to DMSO-treated cells for duplicate experiments. Error bars indicate standard errors of the means. Insets show schematics of reovirus virions and ISVPs. (C) U-2 OS cells were incubated with DMSO, 20 mM NH_4_Cl, or digoxin at the concentrations shown for 1 h prior to adsorption with VSV-eGFP at an MOI of 10 PFU/cell. After 1 h, virus was removed, and cells were incubated with medium containing DMSO or inhibitor for 5 h. Cells were incubated with Hoechst stain to detect nuclei and imaged by fluorescence microscopy. Results are presented as percentages of infected cells normalized to DMSO-treated cells for triplicate experiments. Error bars indicate the standard errors of the means. *, *P* < 0.05, **, *P* < 0.01, and ***, *P* < 0.001, in comparison to DMSO, as determined by ANOVA followed by Tukey’s post hoc test.

To determine whether digoxin blocks infection by other strains of CHIKV as well as related alphaviruses, we assessed the effect of digoxin treatment on infection by CHIKV strains SL15649 and 181/25, RRV strain T48, and SINV strain TRSB. U-2 OS cells were treated with DMSO, 5-NT, or digoxin for 1 h prior to adsorption with CHIKV, RRV, or SINV at a multiplicity of infection (MOI) of 1, 10, or 5 PFU/cell, respectively, to adjust for infectivity differences in these cells. At 6 h postadsorption, cells were scored for infection by indirect immunofluorescence (Fig. 6A). Digoxin treatment significantly diminished infection by all strains tested, with EC_50_s of 108.9 nM for CHIKV strain SL15649, 100.9 nM for CHIKV strain 181/25, 126.5 nM for RRV, and 198.9 nM for SINV.

To determine whether digoxin displays inhibitory effects against diverse virus families, we assessed the capacity of the drug to inhibit mammalian reovirus, a nonenveloped, double-stranded RNA virus. Human brain microvascular endothelial cells (HBMECs) were treated with DMSO, 5-NT, or digoxin prior to adsorption with reovirus virions or infectious subvirion particles (ISVPs). ISVPs are reovirus disassembly intermediates formed following endocytosis and cleavage of the viral outer capsid by intracellular cathepsins or *in vitro* following protease treatment (34). ISVPs bind to cell surface receptors and internalize at the plasma membrane, bypassing the disassembly requirements of virions, including acidic pH and protease activity (34–37). At 20 h postadsorption with either reovirus virions or ISVPs, cells were scored for infection by indirect immunofluorescence (Fig. 6B). Treatment with digoxin impaired infection by both virions (Fig. 6B, left) and ISVPs (Fig. 6B, right), with EC_50_s of 133.9 nM and 434.7 nM, respectively. Decreased infectivity of ISVPs following digoxin treatment suggests that digoxin inhibits reovirus infection at one or more replication steps following internalization and disassembly. We also tested the capacity of digoxin to inhibit VSV, an enveloped, negative-sense RNA virus (Fig. 6C). Digoxin treatment similarly inhibited infection by VSV, with an EC_50_ of 238.7 nM. These data indicate that digoxin inhibits infection by plus-strand, minus-strand, and double-stranded RNA viruses.

### Digoxin impairs CHIKV infection at postentry steps.

**FIG 7:**
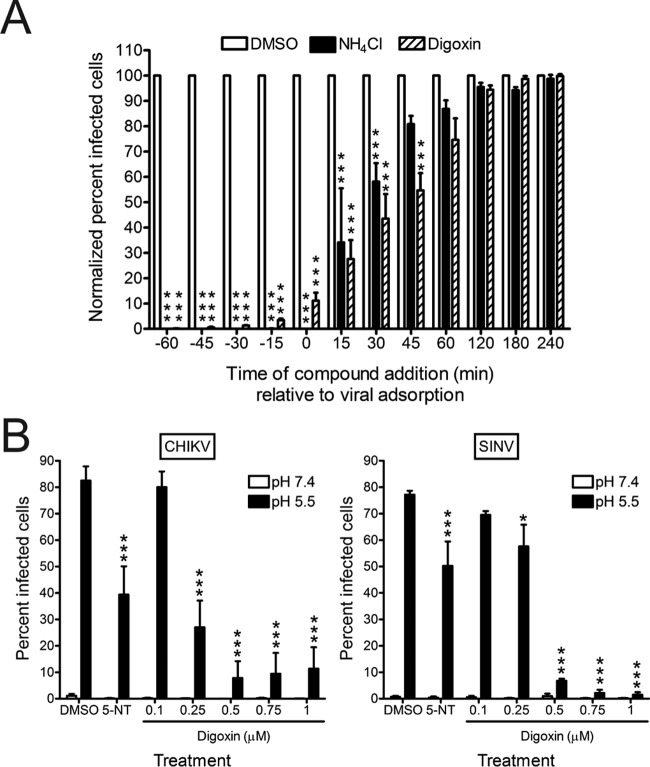
Digoxin inhibits CHIKV at postentry steps of the replication cycle. (A) U-2 OS cells were incubated with DMSO, 20 mM NH_4_Cl, or 1 µM digoxin prior to (−60 to −15 min), during (0 to +45 min), or after (+60 to +240 min) adsorption with CHIKV 181/25 at an MOI of 5 PFU/cell for 1 h. Cells were incubated in the presence or absence of inhibitors for 5 h and scored for infection by indirect immunofluorescence. Results are presented as percentages of infected cells normalized to DMSO-treated cells for triplicate experiments. Error bars indicate standard errors of the means. (B) U-2 OS cells were incubated with DMSO, 10 µM 5-NT, or digoxin at the concentrations shown for 1 h prior to adsorption with CHIKV strain 181/25 (left) or SINV strain TRSB (right) at an MOI of 100 PFU/cell at 4°C for 1 h. Unbound virus was removed, and cells were treated at 37°C for 5 min with either acidic medium (pH 5.5 [black bars]) to trigger viral fusion at the plasma membrane or neutral medium (pH 7.4 [white bars]) as a control. Cells were incubated at 37°C for 18 h with medium containing DMSO or inhibitor and NH_4_Cl to block subsequent rounds of infection. Cells were scored for infection by indirect immunofluorescence. Results are presented as percentages of infected cells for triplicate experiments. Error bars indicate standard errors of the means. *, *P* < 0.05, and ***, *P* < 0.001, in comparison to DMSO, as determined by ANOVA followed by Tukey’s post hoc test.

We next sought to define steps in CHIKV replication blocked by digoxin. To determine the temporal window during which digoxin acts to inhibit CHIKV infection, U-2 OS cells were treated with DMSO or 1 µM digoxin at 15-min intervals for 1 h prior to adsorption or at 15- or 60-min intervals for 4 h after adsorption. As a control for inhibition of CHIKV entry, 20 mM NH_4_Cl was added at the same intervals to block acidification of endocytic compartments. Cells were fixed at 6 h postadsorption and scored for infection by indirect immunofluorescence (Fig. 7A). Maximal impairment of CHIKV infection by digoxin was achieved when digoxin was added 60 min prior to adsorption. The magnitude of inhibition gradually decreased when the drug was added at later times, with only negligible effects observed when added 120 min postadsorption. CHIKV bypassed digoxin inhibition with kinetics similar to the bypass of NH_4_Cl inhibition, suggesting that digoxin restricts CHIKV infection at early steps in the replication cycle. Alphavirus nonstructural proteins and double-stranded RNA (dsRNA) accumulate at the plasma membrane of infected cells to form replication complexes, as early as 45 min postadsorption, and RNA synthesis can be detected by 1 h postadsorption (28, 38). Thus, digoxin may inhibit these or earlier steps in CHIKV replication.

To determine whether digoxin blocks CHIKV infection by inhibiting viral entry (attachment, internalization, and membrane fusion), cells were treated with DMSO, 5-NT, or digoxin for 1 h prior to adsorption with CHIKV or SINV at 4°C to prevent internalization. At 1 h postadsorption, cells were exposed to acidic medium (pH 5.5) to trigger viral fusion at the plasma membrane or neutral medium (pH 7.4) as a control. Fused and unfused cells were incubated with drug and NH_4_Cl at 37°C to block subsequent rounds of replication and scored for infection at 18 h postadsorption by indirect immunofluorescence (Fig. 7B). Triggering fusion at the plasma membrane partially bypassed inhibition of CHIKV and SINV by 5-NT treatment, which inhibits viral entry steps (30). However, fusion at the plasma membrane did not bypass digoxin-mediated inhibition of CHIKV or SINV, except at the lowest concentrations used. The failure of fusion at the plasma membrane to bypass digoxin-mediated inhibition was not due to decreased virus attachment, as the percentage of digoxin-treated cells bound by CHIKV was similar to that of DMSO-treated cells (see Fig. S1 in the supplemental material). Viral titers were similarly decreased following electroporation of digoxin-treated cells with CHIKV RNA relative to those following electroporation of DMSO-treated cells (see Fig. S2 in the supplemental material). Together, these data suggest that CHIKV replication is impeded by digoxin at one or more postentry steps of the viral replication cycle.

### Polymorphisms observed in digoxin-resistant CHIKV populations.

**FIG 8:**
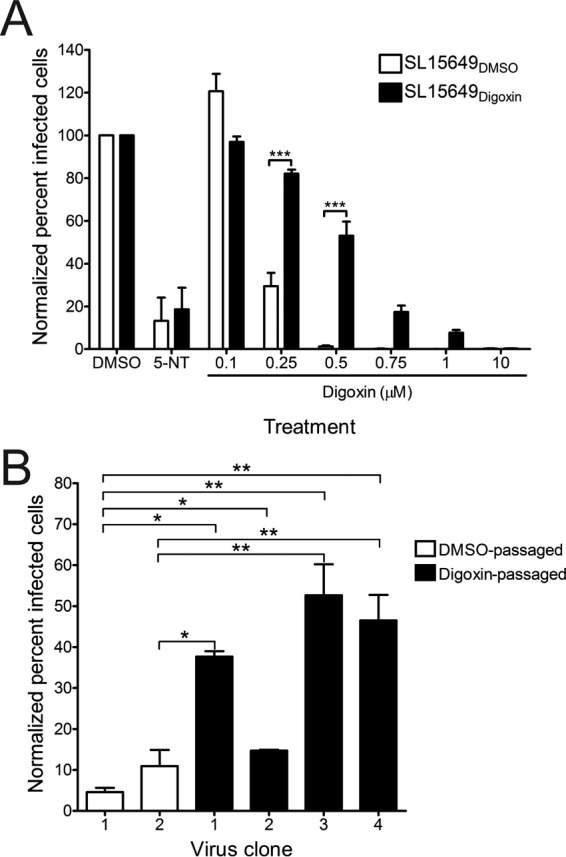
Passage of CHIKV in the presence of digoxin enriches for drug-resistant viruses. U-2 OS cells were incubated with DMSO, 10 µM 5-NT, or increasing concentrations of digoxin for 1 h prior to adsorption with CHIKV stocks that had been passaged 14 times in the presence of either DMSO (SL15649_DMSO_) or digoxin (SL15649_Digoxin_) at an MOI of 5 PFU/cell. After 1 h, virus was removed, and cells were incubated with medium containing DMSO or inhibitor for 5 h. Cells were stained with CHIKV-specific antiserum and DAPI to detect nuclei and imaged by fluorescence microscopy. Results are presented as percentages of infected cells normalized to DMSO-treated cells. Error bars indicate standard errors of the means. (B) U-2 OS cells were incubated with DMSO or 500 nM digoxin for 1 h prior to adsorption at an MOI of 5 PFU/cell with virus clones that were plaque purified from either the SL15649_DMSO_ or SL15649_Digoxin_ stock. After 1 h, virus was removed, and cells were incubated with medium containing DMSO or digoxin for 5 h. Cells were stained with CHIKV-specific antiserum and DAPI to detect nuclei and imaged by fluorescence microscopy. Results are presented as percentages of infected cells normalized to DMSO-treated cells for duplicate experiments. Error bars indicate standard errors of the means. *, *P* < 0.05, **, *P* < 0.01, and ***, *P* < 0.001, in comparison to DMSO-treated or DMSO-passaged virus-infected cells as determined by ANOVA followed by Tukey’s post hoc test.

To enhance an understanding of mechanisms underlying the digoxin-mediated restriction of CHIKV infection, we passaged CHIKV in cells treated with either DMSO or digoxin to select digoxin-resistant viruses. U-2 OS cells were adsorbed with CHIKV strain SL15649 for 1 h and incubated with medium containing either DMSO or 100 nM digoxin until comparable cytopathic effect (CPE) was observed. Supernatants from infected cells were used to inoculate fresh cells, and this process was repeated with increasing concentrations of digoxin until CPE was observed with doses 5 times the EC_50_ in U-2 OS cells. To test whether supernatants of digoxin-treated cells contained drug-resistant viruses, U-2 OS cells were pretreated with DMSO, 5-NT, or digoxin prior to adsorption with passage 14 supernatants from DMSO-treated (SL15649_DMSO_) and digoxin-treated (SL15649_Digoxin_) cells. Infected cells were scored for infection by indirect immunofluorescence at 6 h postinfection (Fig. 8A). Treatment of cells with 5-NT prevented infection by both SL15649_DMSO_ and SL15649_Digoxin_ supernatant stocks. In contrast, treatment of cells with 500 nM digoxin completely inhibited infection by SL15649_DMSO_ (EC_50_ of 198.8 nM), but this dose resulted in only an ~50% inhibition of infectivity by SL15469_Digoxin_ (EC_50_ of 521.4 nM). These data suggest that viral variants selected during serial passage in digoxin-treated cells are less susceptible to the inhibitory effects of digoxin.

To determine the genetic basis for digoxin resistance, we isolated virus clones from the SL15649_DMSO_ and SL15649_Digoxin_ supernatant stocks by plaque purification and assessed the sensitivity of these virus clones to digoxin treatment (Fig. 8B). In general, virus clones isolated from the SL15649_Digoxin_ stock were more resistant to digoxin-mediated inhibition than those isolated from the SL15649_DMSO_ stock, but one clone (SL15649_Digoxin_ clone 2) was sensitive to digoxin treatment like that of the SL15649_DMSO_ clones. To identify polymorphisms displayed by these virus clones, we determined the full-length nucleotide sequences of each clone (Table 1). Sequence analysis identified three unique, nonsynonymous mutations in virus clones from DMSO-treated cells and six unique, nonsynonymous mutations in virus clones from digoxin-treated cells. The E2 S159R polymorphism was identified in virus clones from both DMSO- and digoxin-treated cells and likely was selected as a consequence of cell culture adaptation and increased glycosaminoglycan dependence (39). However, the unique mutations identified in virus clones from digoxin-treated cells were contained in the nonstructural open reading frame. Although the majority of the mutations were in nsP3, these mutations did not segregate completely with digoxin resistance, as digoxin-sensitive SL15649_Digoxin_ clone 2 also encoded these changes. Instead, a mutation in the RNA-dependent RNA polymerase (RdRp), nsP4 V209I, was the only coding change that differed between SL15649_Digoxin_ clone 2 (sensitive) and clones 3 and 4 (resistant). All 290 CHIKV strain sequences available in the NIAID ViPR database encode a valine at residue 209 in nsP4 (40), suggesting that this residue or region of nsP4 serves an important, conserved function in CHIKV replication. These findings indicate that multiple mutations are selected during passage of CHIKV in digoxin-treated cells. Furthermore, selection of mutations in the nonstructural proteins, particularly nsP4 V209I, suggests that digoxin inhibits CHIKV replication by disrupting RNA synthesis or replication complex formation.

**TABLE 1:** Polymorphisms identified by serial passage of CHIKV in cells treated with either DMSO or digoxin

Virus clone^a^	Digoxin sensitivity	Viral gene product	Polymorphism(s)^b^
SL15649_DMSO_			
Clone 1	Sensitive	E2	H170L
Clone 2	Sensitive	nsP1	P249L
		nsP3	S511R
		E2	S159R
SL15649_Digoxin_			
Clone 1	Resistant	nsP2	T757A
		nsP3	D31N, P464L, Stop524_L525del
		E2	S159R
Clone 2	Sensitive	nsP3	D31N, Stop524C
		E2	S159R
Clone 3	Resistant	nsP3	D31N, Stop524C
		nsP4	V209I
		E2	S159R
Clone 4	Resistant	nsP3	D31N, Stop524C
		nsP4	V209I
		E2	S159R

a Virus titers in supernatants from infected DMSO- or digoxin-treated cells were determined by plaque assay, and viruses from individual plaques were amplified using U-2 OS cells.

b RNA isolated from supernatants of infected U-2 OS cells was used as a template for cDNA synthesis with random hexamers. Overlapping fragments covering the genome were amplified using CHIKV-specific primers and sequenced by Sanger sequencing.

## DISCUSSION

CHIKV has reemerged to cause epidemics of fever, rash, and arthritis throughout Africa, South and Southeast Asia, and the Americas. The rapid, mosquito-borne transmission of CHIKV has resulted in millions of cases of CHIKV disease in the last decade alone. The high attack rate and global significance of CHIKV infection warrant development of CHIKV-specific therapeutics and vaccines. Thus far, none are available.

To enhance an understanding of the host factors required for CHIKV replication, we screened a library of chemical compounds for the capacity to diminish or enhance CHIKV infection in U-2 OS cells. We identified digoxin, a cardiac glycoside, as a host species-specific inhibitor of CHIKV replication. We discovered that digoxin impedes CHIKV infection at postentry steps of the replication cycle via antagonism of the sodium-potassium ATPase. Passage of CHIKV in digoxin-treated cells selected multiple mutations in the nonstructural proteins, and one particular mutation, nsP4 V209I, segregated with digoxin sensitivity. Taken together, these findings indicate that a functional sodium-potassium ATPase is required for CHIKV infection.

Our results demonstrate that CHIKV inhibition by digoxin occurs at an early but postentry step in the viral replication cycle. The temporal window in which digoxin inhibition occurs mirrors that of ammonium chloride, which supports an early block to CHIKV infection (Fig. 7A). However, fusion of CHIKV or SINV at the plasma membrane in an attempt to bypass the digoxin-mediated defect was not sufficient to restore viral replication to levels in untreated cells (Fig. 7B). In an analogous case, proteolytic cleavage of reovirus virions to form ISVPs *in vitro* overcame restriction by an entry inhibitor, 5-NT, but was insufficient to completely circumvent inhibition by digoxin. ISVPs are thought to enter cells at the plasma membrane and, therefore, are not susceptible to inhibition by compounds that inhibit reovirus internalization or disassembly (35, 36, 41). Selection of multiple mutations in CHIKV nonstructural proteins following passage of virus in digoxin-treated cells also points to inhibition of postentry viral replication steps as a mechanism of digoxin action. Although the majority of these mutations were selected in nsP3, a protein essential for formation of CHIKV replication complexes and modulation of host stress responses (42–46), these mutations also were observed in digoxin-sensitive variants (SL15649_Digoxin_ clone 2). In otherwise isogenic viruses (SL15649_Digoxin_ clones 2 to 4), the nsP4 V209I polymorphism was sufficient to confer resistance to digoxin treatment, but the digoxin-resistant phenotype may depend on the presence of the nsP3 Stop524C mutation to enhance levels of nsP4, suggesting that one or more functions of nsP4 are impaired by digoxin. However, additional combinations of mutations also may produce a digoxin-resistant virus (SL15649_Digoxin_ clone 1). We conclude that individual or combinatorial substitutions within genes encoding nonstructural proteins can restore replication efficiency to bypass digoxin inhibition, with the substitution in nsP4 playing a pivotal role.

Inhibition of the sodium-potassium ATPase disrupts ion transport and alters many cellular biosynthetic, signaling, and vesicular sorting pathways (47). Although the precise alterations by which digoxin restricts CHIKV infection are not known, we envision two possible mechanisms. First, CHIKV may require a specific ion composition to complete discrete steps in the viral replication cycle or coordinate the functions of specific viral proteins. Evidence for such an ion requirement is supported by the function of the alphavirus 6K protein in ion channel formation and the sensitivity of alphavirus particle maturation to the ionic strength of the culture medium (48, 49). The ion channels formed by 6K are selective for Na^+^, K^+^, and Ca^2+^ ions, which are specifically altered during cardiac glycoside treatment (48). In support of this possibility, we found that addition of extracellular potassium during digoxin treatment restored infectivity to levels observed for DMSO-treated cells (Fig. 4B). Second, digoxin may induce cellular stress responses to impede CHIKV replication. Cardiac glycoside treatment stimulates interactions between the sodium-potassium ATPase and the inositol 1,4,5-trisphosphate receptor (Ins[1,4,5]P_3_R) to elicit calcium oscillations and activate calcium-dependent transcription factors, such as nuclear transcription factor κB (NF-κB) (50, 51). Activation of NF-κB enhances the expression of gene products involved in apoptosis and innate immune responses that promote an antiviral state that could ultimately restrict CHIKV infection (52). Although digoxin treatment induced modest activation of NF-κB (see Fig. S3 in the supplemental material), this increase was not statistically significant, suggesting that other calcium-dependent host molecules contribute to the restriction of CHIKV by digoxin. For example, it is possible that formation of CHIKV replication complexes and functions of specific viral proteins require precise spatial and temporal regulation of certain ion concentrations. Digoxin may directly perturb these viral activities to impede replication or trigger host stress responses that, in turn, accomplish the same effect. Digoxin-resistant viral variants may overcome this impairment by altering residues in the nonstructural proteins, including nsP4 V209I, to enhance ion-regulated functions of these proteins.

The complexity of cardiac glycoside-mediated effects on cells may contribute to multiple mechanisms of CHIKV inhibition, which would make it difficult to select digoxin-resistant mutants. Indeed, passage of CHIKV in digoxin-treated cells led to development of drug resistance only after approximately 14, 72-h passages. Digoxin is currently FDA approved for the treatment of congestive heart failure and cardiac arrhythmias, but clinically significant toxicity of digoxin precludes its widespread use (53–55). In patients treated with digoxin, acceptable serum concentrations range between 1 and 2.5 nM, concentrations appreciable lower than those required to inhibit CHIKV *in vitro*. Therefore, additional work is required to develop drugs that more selectively target steps within this pathway to limit toxicity. In this regard, less toxic derivatives of digoxin are effective in inhibition of T_H_17 cell differentiation in the treatment of autoimmune diseases (56). The capacity of ouabain to inhibit CHIKV infection suggests that these analogous compounds also would diminish CHIKV infection in a similar manner.

Identification of CHIKV-specific therapeutics requires an improved understanding of CHIKV replication and pathogenesis. Findings presented in this report suggest that the sodium-potassium ATPase serves an essential function in CHIKV infection. Antagonism of this ion transporter with digoxin inhibits CHIKV infection of human cells. Digoxin inhibits one or more postentry steps of the CHIKV replication cycle as evidenced by time-of-addition and fusion-bypass experiments. Further studies to delineate mechanisms by which blockade of the sodium-potassium ATPase impedes CHIKV infection will illuminate host factors and pathways required for CHIKV replication. Such factors may serve as additional drug targets to ameliorate CHIKV disease.

## MATERIALS AND METHODS

### Cells, chemical inhibitors, antibodies, and plasmids.

U-2 OS cells were maintained in McCoy’s 5A medium (Gibco) supplemented to contain 10% fetal bovine serum (FBS [Gibco]). Primary HSFs were provided by James W. Thomas (Vanderbilt University) and cultivated as described previously (57). ST2 cells were provided by Julie A. Sterling (Vanderbilt University) and maintained in RPMI 1640 medium supplemented to contain 10% FBS. C2C12 cells were provided by David M. Bader (Vanderbilt University) and maintained in Dulbecco’s modified Eagle’s medium (DMEM) supplemented to contain 10% FBS. Baby hamster kidney (BHK-21), C6/36, and Vero cells were cultivated as described previously (58). HBMECs were provided by Kwang Sik Kim (Johns Hopkins University) and cultured in RPMI 1640 medium as described previously (59). L929 cells were maintained in Joklik’s minimum essential medium supplemented to contain 5% FBS. All media for cell maintenance were supplemented to contain 2 mM l-glutamine (Gibco), 100 U/ml penicillin, 100 µg/ml streptomycin (Gibco), and 25 ng/ml amphotericin B (Sigma).

Bafilomycin A1 (Sigma), digoxin (Sigma), 5-NT oxalate (Tocris), ouabain octahydrate (Sigma), and STS (Cell Signaling Technology) were resuspended in DMSO. Polyclonal antisera obtained from ATCC were used for CHIKV (VR-1241AF), RRV (VR-1246AF), and SINV (VR-1248AF) infectivity assays. Reovirus-specific polyclonal antiserum (60) was used for reovirus infectivity assays.

### Biosafety.

Experiments involving the generation and testing of CHIKV SL15649 replicon particles and replication-competent CHIKV were conducted in a certified biological safety level 3 (BSL3) facility in biological safety cabinets with protocols approved by the Vanderbilt University Department of Environment, Health, and Safety and the Vanderbilt Institutional Safety Committee.

### Generation of CHIKV replicon particles.

The three-plasmid CHIKV SL15649 replicon system was used as described previously (61). Plasmids encoding CHIKV nonstructural proteins and eGFP, capsid protein, and the envelope glycoproteins (E3 to E1) were linearized and transcribed *in vitro* using mMessage mMachine SP6 transcription kits (Ambion). BHK-21 cells were electroporated with viral RNAs generated from the three plasmids and incubated at 37°C for 24 h. Supernatants containing replicon particles were collected from electroporated cells, clarified by centrifugation, and stored at −80°C. Replicon particles were tested for propagation-competent recombinant virus by serial passage of replicon stocks on monolayers of Vero cells. Stocks were removed from the BSL3 laboratory only if CPE was not detected 72 h after the second passage.

### High-throughput screening of NCC.

U-2 OS cells seeded in 384-well plates (Corning) were treated with DMSO, 100 nM bafilomycin A1, or compounds from the NCC at a concentration of 1 µM using a Bravo automated liquid handling platform (Velocity 11/Agilent) and incubated at 37°C for 1 h. CHIKV SL15649 eGFP-expressing replicon particles were inoculated into wells of treated cells at an MOI of 5 infectious units (IU)/cell and incubated at 37°C for 20 to 24 h. Medium was aspirated using an ELx405 microplate washer (Biotek), and cells were incubated with Hoechst dye to stain nuclei using a Multidrop Combi reagent dispenser (Thermo Scientific). Cells and nuclei were visualized using an ImageXpress Micro XL imaging system. Total cells and infected cells were quantified using MetaXpress software in two fields of view per well. The plate median and median absolute deviation (MAD) were calculated for each well and used to calculate robust *Z* scores with the following equation: *Z* score = [log_2_(% infection) − log_2_(median)]/[log_2_(MAD) × 1.486]. Candidates were considered positive if the robust *Z* score was ≤−2 or ≥2 in at least two of three independent replicates.

### Generation of virus stocks.

The CHIKV 181/25 and SL15649 infectious clone plasmids were generated as described previously (62). Plasmids containing the full-length cDNA sequences of RRV strain T48 (pRR64) and SINV strain AR339 (pTRSB) were generated as described previously (63, 64). CHIKV, RRV, and SINV infectious clone plasmids were linearized and transcribed *in vitro* using mMessage mMachine SP6 transcription kits. BHK-21 cells were electroporated with viral RNA and incubated at 37°C for 24 h. Supernatants containing progeny virus were collected from electroporated cells, clarified by centrifugation, and stored at −80°C. Viral titers were determined by plaque assay using Vero or BHK-21 cells. All experiments with CHIKV SL15649 virus were performed using BSL3 conditions.

Reovirus strain T1L M1 P208S (65) was generated using plasmid-based reverse genetics (66). Purified virions were prepared as described previously (67). The reovirus particle concentration was determined from the equivalence of 1 U of optical density at 260 nm to 2.1 × 10^12^ particles (68). Viral titers were determined by plaque assay using L929 cells (66). ISVPs were generated by treating virion particles with α-chymotrypsin (Sigma) as described previously (37).

VSV-eGFP was provided by Sean Whelan (Harvard University) and propagated on BHK-21 cells as described previously (69). Viral titers were determined by plaque assay using Vero cells.

### CHIKV, RRV, and SINV infectivity assays.

Vehicle- or compound-treated U-2 OS, HSF, ST2, C2C12, and C6/36 cells seeded in 96-well plates (Costar) were adsorbed with CHIKV, RRV, or SINV diluted in virus diluent buffer (VDB) (RPMI 1640 medium with 25 mM HEPES and 1% FBS) at various MOIs at 37°C (U-2 OS, HSF, ST2, and C2C12) or 28°C (C6/36) for 1 h. The inoculum was removed, complete medium containing DMSO or compound was added, and cells were incubated at 37°C or 28°C for an additional 5 h. Cells were fixed with ice-cold methanol, washed with PBS, and incubated with PBS containing 5% FBS and 0.1% Triton X-100 (TX) at room temperature for 1 h. Cells were incubated with CHIKV-, RRV-, or SINV-specific polyclonal antiserum (1:1,500) in PBS with FBS and TX at 4°C overnight. Cells were washed three times with PBS and incubated with Alexa Fluor 488-labeled anti-mouse IgG (1:1,000) in PBS with FBS and TX at room temperature for 2 h. Cells also were incubated with 4′,6-diamidino-2-phenylindole (DAPI; Invitrogen) to stain nuclei. Cells and nuclei were visualized using an ImageXpress Micro XL imaging system in four fields of view per well. Percentage of infectivity was determined by dividing the number of virus-infected cells by the total number of cells per field of view. CHIKV infectivity following digoxin treatment was determined in the presence of increasing concentrations of sodium and potassium by pretreating U-2 OS cells with vehicle or digoxin diluted in complete medium or in medium supplemented with NaCl or KCl at 37°C for 1 h. Pretreated cells were adsorbed with CHIKV strain 181/25 at an MOI of 5 PFU/cell at 37°C for 1 h. The inoculum was removed, complete medium or ion-supplemented medium containing DMSO or digoxin was added, and cells were incubated at 37°C for an additional 5 h. Cells were fixed, stained by indirect immunofluorescence to detect CHIKV antigen and nuclei, and visualized as described above.

### Expression of gene transcripts by RT-PCR.

RNA was isolated from U-2 OS and ST2 cells using a PureLink RNA minikit (Ambion). cDNA was prepared with the SuperScriptIII first strand kit (Invitrogen) with random hexamers to prime cDNA synthesis and used for PCR amplification by KOD polymerase with primers specific for the human or murine α1 and α3 isoforms of the sodium-potassium ATPase and GAPDH as a control (for primer sequences, see Table S1 in the supplemental material). Reaction products were resolved by electrophoresis in 1% agarose gels (Life Technologies).

### Assessment of cell viability.

U-2 OS cells seeded in 60-mm-diameter dishes were incubated with DMSO, 10 µM STS as an inducer of apoptosis, or increasing concentrations of digoxin at 37°C for 6 h. Cells were washed with fluorescence-activated cell sorter (FACS) buffer (PBS with 2% FBS) and stained with PI (Sigma). Cell staining was quantified using a BD LSRII flow cytometer and FlowJo software (Tree Star). Alternatively, U-2 OS cells seeded in 96-well plates were incubated with DMSO, STS, or increasing concentrations of digoxin at 37°C for 6 or 24 h. PrestoBlue reagent (Molecular Probes) was added to supernatants of compound-treated cells, and cells were incubated at 37°C for 30 min. Fluorescence as a surrogate for cell viability was quantified using a Synergy H1 plate reader (BioTek).

### Reovirus infectivity assay.

Vehicle- or compound-treated HBMECs seeded in 96-well plates (Costar) were adsorbed with reovirus virions or ISVPs at an MOI of 1,500 particles/cell at room temperature for 1 h. The inoculum was removed, and cells were washed with PBS and incubated with medium containing DMSO or compound at 37°C for 20 h. Cells were fixed with ice-cold methanol, washed with PBS, and incubated with PBS containing 5% bovine serum albumin at room temperature for 15 min. Cells were incubated with reovirus-specific polyclonal antiserum (1:1,000) in PBS with 0.5% TX at 37°C for 30 min. Cells were washed three times with PBS and incubated with Alexa Fluor 488-labeled anti-rabbit IgG (1:1,000) in PBS with 0.5% TX at 37°C for 30 min. Cells also were incubated with DAPI to stain nuclei. Cells and nuclei were visualized using an ImageXpress Micro XL imaging system in four fields of view per well. The percentage of infectivity was determined by dividing the number of virus-infected cells by the total number of cells per field of view.

### VSV infectivity assay.

Vehicle- or compound-treated U-2 OS cells seeded in 96-well plates (Costar) were adsorbed with VSV-eGFP at an MOI of 10 PFU/cell at 37°C for 1 h. The inoculum was removed, complete medium containing DMSO or compound was added, and cells were incubated at 37°C for an additional 5 h. Medium was removed, and cells were incubated with Hoechst dye to stain nuclei. Cells and nuclei were visualized using an ImageXpress Micro XL imaging system in four fields of view per well. The percentage of infectivity was determined by dividing the number of GFP-positive cells by the total number of cells per field of view.

### Fusion-bypass assay.

U-2 OS cells seeded in 96-well plates (Costar) were incubated with DMSO, 5-NT, or increasing concentrations of digoxin at 37°C for 1 h. Compounds were removed, and cells were washed twice with ice-cold binding medium (RPMI 1640 medium with 25 mM HEPES [pH 7.4], 1% FBS, and 20 mM NH_4_Cl) to prevent internalization by endosomal fusion. Cells were adsorbed with CHIKV or SINV diluted in binding medium at 4°C for 1 h, washed twice with binding medium to remove unbound virus, and incubated at 37°C for 5 min with either prewarmed fusion medium (RPMI 1640 medium with 25 mM HEPES, 1% FBS, and 30 mM succinic acid, pH 5.5) to trigger fusion at the plasma membrane or prewarmed binding medium as a control. Medium was removed, cells were incubated with complete medium containing vehicle or inhibitor and 20 mM NH_4_Cl at 37°C, and infection was scored by indirect immunofluorescence at 18 h postadsorption.

### Selection of digoxin-resistant mutants.

U-2 OS cells were adsorbed with CHIKV SL15649 at an MOI of 0.01 PFU/cell in VDB at 37°C for 1 h. Virus was removed, and complete medium containing either DMSO or 100 nM digoxin was added to cells. Cells were incubated at 37°C for 48 to 72 h or until the CPEs were comparable in DMSO- and digoxin-treated cells. Cell culture supernatants were collected, and 0.5 ml was used to inoculate a fresh flask of U-2 OS cells. The remaining supernatant was stored at −80°C. Virus was passaged serially in this manner, gradually increasing the concentration of digoxin until it reached a dose that was 5 times the EC_50_ for the drug in U-2 OS cells.

### Sequence analysis of digoxin-resistant and -sensitive mutants.

Viruses from cell-culture supernatants of DMSO- or digoxin-treated cells were plaque purified using BHK-21 cells, and RNA was isolated using a PureLink RNA minikit. cDNA was prepared with the SuperscriptIII first strand kit with random hexamers to prime cDNA synthesis and subjected to PCR amplification using KOD polymerase with CHIKV-specific primer sets to enable amplification of fragments that collectively encompass the entire viral genome. Amplicons were sequenced using Sanger sequencing (GenHunter, Nashville, TN).

### Statistical analysis.

Mean values for at least duplicate experiments were compared using a one-way analysis of variance (ANOVA) followed by Tukey’s post hoc test (GraphPad Prism). *P* values of <0.05 were considered to be statistically significant.

## SUPPLEMENTAL MATERIAL

Text S1  Supplemental methods. Download Text S1, DOCX file, 0.02 MB

Figure S1  CHIKV attachment to cells is not impaired following digoxin treatment. U-2 OS cells were incubated with DMSO, 10 µM 5-NT, or digoxin at the concentrations shown for 1 h prior to adsorption with CHIKV strain 181/25 at an MOI of 100 PFU/cell at 4°C for 1 h. Unbound virus was removed, cells were stained with CHIKV-specific antiserum, and virus-bound cells were quantified by flow cytometry. Results are presented as percentages of bound cells for duplicate experiments normalized to cell autofluorescence in the absence of CHIKV antiserum. Error bars indicate standard errors of the means. Download Figure S1, TIF file, 0.2 MB

Figure S2  Digoxin inhibits the production of progeny virions from CHIKV RNA-electroporated cells. U-2 OS cells were incubated with DMSO, 10 µM 5-NT, or digoxin at the concentrations shown for 1 h prior to electroporation with CHIKV SL15649 RNA generated *in vitro*. Cells were incubated in complete medium (left) or medium containing DMSO or inhibitor (right). At the times shown, viral titers in culture supernatants were determined by plaque assay. Results are presented as the mean viral titers for triplicate samples. Error bars indicate standard deviations. ***, *P* < 0.001, in comparison to DMSO, as determined by ANOVA followed by Tukey’s post hoc test. Download Figure S2, TIF file, 0.3 MB

Figure S3  Activation of NF-κB following digoxin treatment. U-2 OS cells were transfected with pGL4-3XκB, which expresses firefly luciferase under control of NF-κB, and the control pRL-SV40 plasmid, which constitutively expresses *Renilla* luciferase. At 24 h posttransfection, cells were either adsorbed with CHIKV strain 181/25 at an MOI of 10 PFU/cell for 1 h or treated with the concentrations of digoxin shown. As a positive control, cells were treated with 20 ng/ml TNF-α. Cells were incubated at 37°C for 6 h, and luciferase activity was quantified in cell lysates for triplicate wells. Results are presented as the fold NF-κB activation normalized to mock-treated cells for triplicate experiments. Error bars indicate standard errors of the means. ***, *P* < 0.001, as determined by ANOVA followed by Tukey’s post hoc test. Download Figure S3, TIF file, 0.3 MB

Table S1  Sequences of primers used for detection of human and murine sodium-potassium ATPase subunit transcripts. Table S1, DOCX file, 0.02 MB
