# Supplementary material for: Antagonism of the Sodium-Potassium ATPase Impairs Chikungunya Virus Infection
Source: mBio. 2016 May 24;7(3):e00693-16. doi: 10.1128/mBio.00693-16 (PMC4895112; doi:10.1128/mBio.00693-16)
Supplement: Text S1 — Supplemental methods. Download [file mbo003162824s1.docx]

**SUPPLEMENTAL METHODS**

**CHIKV binding assay.** U-2 OS cells seeded in 60-mm dishes (BD Biosciences) were treated with DMSO, 10 µM 5-NT, or increasing concentrations of digoxin and incubated at 37°C for 1 h. Treated cells were adsorbed in suspension with CHIKV at a multiplicity of infection (MOI) of 100 plaque-forming units (PFU)/cell at 4°C for 1 h. Cells were washed with incomplete medium and PBS and fixed in PBS with 1% electron microscopy (EM)-grade paraformaldehyde (Electron Microscopy Sciences). Cells were washed with fluorescence-activated cell sorting (FACS) buffer (PBS with 2% FBS) and incubated with CHIKV-specific polyclonal antiserum (1:1500) in FACS buffer at 4°C for 30 min. Cells were incubated with Alexa Fluor 488-labeled anti-mouse IgG (1:1000) in FACS buffer at 4°C for 30 min and analyzed using a BD LSRII flow cytometer. Cell staining was quantified using FlowJo software (Tree Star).

**RNA electroporation bypass of virus entry.** U-2 OS cells pretreated with DMSO or inhibitor were removed from the culture dish, washed with PBS, and resuspended to a final concentration of 10^7^ cells/ml. Cells were electroporated with SL15649 RNA generated in vitro. Electroporated cells were seeded into 24-well plates in complete medium or in medium containing DMSO or inhibitor. After incubation at 37°C for various intervals, 10% of the cell culture supernatant was harvested and replaced with fresh medium. Viral titers in culture supernatants were determined by plaque assay using Vero cells.

**NF-κB luciferase reporter assay.** U-2 OS cells seeded in 24-well plates were transfected with 200 ng/well of the pGL4-3XκB plasmid (provided by Albert Baldwin, University of North Carolina), which contains three copies of the NF-κB binding site from the major histocompatibility complex class I promoter, and 50 ng/well of control pRL-SV40 plasmid (Promega) using FuGENE 6 (Promega). After 24 h, cells were adsorbed with CHIKV 181/25 at an MOI of 10 PFU/cell, treated with increasing concentrations of digoxin, or treated with 20 ng/ml of TNF-α as a positive control. Cells were incubated at 37°C for 6 h, and firefly (pGL4-3XκB) and Renilla (pRL-SV40) luciferase activity was quantified using the Dual-Luciferase Assay Kit (Promega) according to the manufacturer’s instructions.
