# Supplementary material for: Antagonism of the Sodium-Potassium ATPase Impairs Chikungunya Virus Infection
Source: mBio. 2016 May 24;7(3):e00693-16. doi: 10.1128/mBio.00693-16 (PMC4895112; doi:10.1128/mBio.00693-16)
Supplement: Table S1 — Sequences of primers used for detection of human and murine sodium-potassium ATPase subunit transcripts. [file mbo003162824st1.docx]

| **Gene** | **Species** | **Forward Primer (5’ 3’)** | **Reverse Primer (5’ 3’)** |
| --- | --- | --- | --- |
| ATP1A1 | Human | CTG TGG ATT GGA GCG ATT CTT | ACC AGT GAG CGA GGA GTT AT |
| ATP1A1 | Mouse | CCT GGA TGA ACT CCA TCG TAA A | TAG CAC TTC GGA TGC CAT AAG |
| ATP1A3 | Human | GAG GTC TGC CGG AAA TAC AA | GAG AAG CAG CCA GTG ATG AT |
| ATP1A3 | Mouse | CAG GGT CTG ACA CAC AGT AAA G | CAC TAT GCC CAG GTA CAG ATT G |
| GAPDH | Human/mouse | CCC ATC ACC ATC TTC CAG | ATG ACC TTG CCC ACA GCC |

Table S1. Sequences of primers used for detection of human and murine transcripts.
